# Broadening the voice of science: Promoting scientific communication in the undergraduate classroom

**DOI:** 10.1002/ece3.3501

**Published:** 2017-10-24

**Authors:** Lauren A. Cirino, Zachary Emberts, Paul N. Joseph, Pablo E. Allen, David Lopatto, Christine W. Miller

**Affiliations:** ^1^ Entomology & Nematology Department University of Florida Gainesville FL USA; ^2^ Department of Biology University of Florida Gainesville FL USA; ^3^ Department of Psychology Grinnell College Grinnell IA USA

**Keywords:** classroom‐based research, CURE, nature of science, oral communication, science skill development, undergraduate research

## Abstract

Effective and accurate communication of scientific findings is essential. Unfortunately, scientists are not always well trained in how to best communicate their results with other scientists nor do all appreciate the importance of speaking with the public. Here, we provide an example of how the development of oral communication skills can be integrated with research experiences at the undergraduate level. We describe our experiences developing, running, and evaluating a course for undergraduates that complemented their existing undergraduate research experiences with instruction on the nature of science and intensive training on the development of science communication skills. Students delivered science talks, research monologues, and poster presentations about the ecological and evolutionary research in which they were involved. We evaluated the effectiveness of our approach using the CURE survey and a focus group. As expected, undergraduates reported strong benefits to communication skills and confidence. We provide guidance for college researchers, instructors, and administrators interested in motivating and equipping the next generation of scientists to be excellent science communicators.

## 1 INTRODUCTION

Antibiotics kill viruses (Collett, Pappas, Evans, & Hayden, 1999), vaccines cause autism (Ruiz & Bell, 2014), and global climate change is a conspiracy (van der Linden, 2015). These, among many others, are huge misconceptions of scientific knowledge that are perpetuated by the fast flow of information through the internet. Disseminating inaccurate information has become a normal occurrence as avenues for information on the Internet have expanded (Del Vicario et al., 2016). Now, it is more crucial than ever for scientists of all levels to learn how to effectively communicate their science and to do so frequently through multiple media. The way in which scientific information is discussed is important for helping the lay public make decisions about their health care, the environment, public funding of scientific research, and the quality of food that they consume. Of course, communication of scientific findings is also essential among scientists themselves to enable science to proceed in new and innovative directions (Osterhaus & Vanlangendonck, 2015).

Training future generations of scientists in effective communication is imperative. Often, no formal training in science communication is available for undergraduates majoring in scientific fields (Coil, Wenderoth, Cunningham, & Dirks, 2010 but see Mercer‐Mapstone & Kuchel, 2015). Thus, undergraduate students more commonly gain these skills by happenstance (e.g. they join a laboratory that makes science communication a priority) or by seeking formal science communication training independently (Coil et al., 2010). High‐quality science communication training should be the norm, not the exception for young scientists. Emphasis should be placed on training young scientists in how to effectively communicate research to different audiences, so that accurate dissemination of scientific information is shared with both the public and the scientific community (Besley, Dudo, & Storksdieck, 2015; Brownell, Price, & Steinman, 2013; Kuehne et al., 2014; Pace et al., 2010).

Engaging undergraduates in authentic research experiences is a national priority in STEM (Handelsman & Brown, 2016). Benefits for undergraduates include enhanced professional skills, thinking and working like a scientist, confidence in research skills, and an increased interest in a STEM career (Russell, Hancock, & McCullough, 2007; Seymour, Hunter, Laursen, & Deantoni, 2004). Yet, despite these benefits, a frequent problem is that undergraduates can complete a semester of research without understanding the importance or broader context of the work in which they engaged (Coil et al., 2010). When students are not encouraged to think critically about the value and context of their research, they may also lack the ability to communicate the value of their work to their peers, family, and community. According to Mulder et al. (2008) and Chan (2011), research training is greatly enhanced by training in science communication. Many undergraduate research opportunities have incorporated science communication into the curriculum (Miller, Hamel, Holmes, Helmey‐Hartman, & Lopatto, 2013; Sarmah et al., 2016; Seymour et al., 2004), yet these approaches are not common and also not comprehensive. We propose that a valuable strategy to improving science literacy is training students on how to orally communicate science to multiple audiences concurrent with and throughout authentic research experiences. Thus, we direct this manuscript to researchers in the biological sciences that involve undergraduates in their research programs. With relatively little effort, PIs and their research teams can engage their undergraduate researchers in science communication training, to the benefit of the entire research laboratory and beyond.

In this manuscript, we describe our experiences developing, teaching, and evaluating a course focused on elevating science oral communication skills in advanced undergraduates. Together, with a teaching team, we recruited undergraduates that had at least one prior semester of research experience. The goal was to help these students become skillful in orally communicating science to scientists and the public while simultaneously thinking more critically about their research projects. This course incorporated elements relevant to the development of science oral communication skills, including materials and discussions about the processes of science, science ethics, and science in the media. Further, we revisited and reinforced student understanding of ecology and evolutionary biology to help them gain deeper context for their ongoing laboratory research projects (e.g. Cirino & Miller, 2017; Emberts, St Mary, & Miller, 2016; Joseph, Sasson, Allen, Somjee, & Miller, 2016; Miller, McDonald, & Moore, 2016). Activities specifically focused on science oral communication involved (i) the presentation of primary scientific literature to peers, (ii) the development of a research monologue for the public, and (iii) the construction and presentation of a technical poster based on their current undergraduate research. The time investment of the PI and four graduate students involved in teaching this course was low (approximately 2 hr per person per week; see S1) relative to the strong learning gains reported by the undergraduates involved. Our main goal in this manuscript is to provide an example of how to invest in the development of a new generation of scientists equipped with excellent science oral communication skills while maintaining the momentum of authentic research in the PI's laboratory. We provide a description of our experiences, a report of student learning gains, benefits to instructors, and a practical guide for others to use when building one of these courses.

## 2 COURSE DESIGN AND STUDENT RECRUITMENT

The teaching team, a primary investigator and four graduate students from a single research laboratory, co‐designed this course for undergraduate students that already had one semester of authentic research experience. These students continued in research within and outside of the classroom (Table 1; Auchincloss et al., 2014). Six of the seven students in this course were involved in evolutionary ecology and behavior research in the laboratory of the course instructors, with one student involved in fungal ecology research under a faculty member not involved with this course. This course was inspired by the classroom undergraduate research experiences (CURE) courses (Lopatto, 2008a). The components of a CURE course, collaboration, iteration, discovery, implementation of scientific practices, and student contributions to building on knowledge, were achieved through classroom support and co‐instructor research mentorship (Auchincloss et al., 2014; Brownell & Kloser, 2015). We focus here primarily on the development of oral communication skills, even though all five categories of a CURE were met during the semester (see S1 for more course details).

**Table 1:** Course elements for the CURE course

Course element	Topic	Training location
Foundational topics and materials	What is science? Evolutionary ecology & behavior Science ethics	Classroom
Science communication training	Scientific Literacy & Communication The Research Talk for Peers The 1‐min Monologue for the Public The Research Poster for the Scientific Community	Classroom
Ongoing authentic research	Data analysis training Laboratory techniques training Authentic research	Classroom and undergraduate student's respective laboratory

We offered this course at the University of Florida (Gainesville, FL) in the summer of 2015. Students met with co‐instructors once a week for 5 weeks in a two‐and‐a‐half‐hour time block (see S1). Research was accomplished both in the classroom and in the student's respective laboratory. Students committed 20–30 hr of time to research outside of their classroom time. Unlike a traditional lecture‐based science course, our course intentionally wove the students’ own unique experiences with authentic research into the course materials. We encouraged students to draw upon their own experiences to make connections with scientific theory and how science is portrayed in the public sphere. Further, we used students’ research as the material for their science communication training (Box 1).

Box 1 **Applying science communication curriculum to an undergraduate classroom**

1 *Find the instructors and provide them an excellent opportunity for career development* Identify graduate students, postdoctoral researchers, and even experienced undergraduates with similar commitments to excellence in science education. Designate specific meeting dates and times for collaboration prior to and throughout the class. Once your teaching team is assembled, collaborate to design and teach the course, assigning tasks by interest and skill level. By involving advanced students in this process, you will provide them with an excellent training opportunity in teaching. If you plan on involving students outside of your laboratory, consider having someone involved with the course that is affiliated with those laboratories. This approach will help answer research‐specific questions and contribute to students feeling like they are part of a learning community.

*Determine the science communication assignments* Identify two to three science communication assignments for the class based on the length of the class, the amount of time each assignment will take, and the skill level of your students. Determine how each assignment will be taught, who will teach it, and how it will be graded (see S2 and S3). There should be other assignments in this class to support the science communication curriculum such as investigating specific research topics using the primary literature or discussing what it means to be a scientist.

*Provide support for student science communication learning* Give students the opportunity to practice their science communication with each other and the teaching team. Provide constructive feedback (e.g. presentation rubric; see S2 and S3) before their final presentation of each science communication assignment.

*Showcase undergraduate research* Have your undergraduate students present their research in a school‐wide research forum or conference, video record your students presenting their research to the public, or have your students present their research to high school or middle school life science classes.

Our course used a flipped classroom approach (Lage, Platt, & Treglia, 2000) to provide training in fundamental nature of science topics (Table 1; Lederman, 2007). Throughout, we trained students in three forms of science communication: a research talk, a 1‐min research monologue, and a research poster.

### 2.1 The research talk for peers

Students worked in pairs to critically evaluate a manuscript published in the primary scientific literature and then crafted a professional 8‐min PowerPoint presentation of the introduction, methods, results, and conclusion. Students were given a presentation rubric ahead of time to help them develop this presentation (see S2). Students had a mandatory meeting with one of the co‐instructors to share a practice version of the talk and gather feedback prior to the final presentation. After each final presentation, the students provided the class with questions to stimulate meaningful discussions among their peers as relevant to the manuscript being addressed. Co‐instructors graded students during the presentations using a standardized rubric in which points were given for delivery, slide preparation and content, and evidence of a rehearsed talk (see S2).

### 2.2 The 1‐min research monologue for the public

Students were asked to draw on their knowledge of their ongoing research to prepare a 1‐min research monologue targeting a general audience of a middle‐school reading level (Kutner, Greenberg, & Baer, 2005). Students presented their monologues in a small‐group format where they received feedback from members of the teaching team and their peers (Brownell & Kloser, 2015). After revising their monologues in class, students were filmed giving their presentations. A subset of these videos was posted to the laboratory Web site (http://www.millerlab.net/) as a form of scientific outreach (Brownell & Kloser, 2015). Students received participation points for this assignment as the revision process of this monologue was primarily completed during class time.

### 2.3 The research poster for the scientific community

Students worked with the teaching team to design and present a poster on their laboratory research projects for delivery to peer scientists. A poster presentation rubric was provided at the beginning of the semester to help guide students in designing this presentation (see S3). Students reported on their findings to date or proposed their project in this assignment. Students first presented a draft of their poster halfway through the semester where both their peers and the teaching team provided feedback (Brownell & Kloser, 2015). Students presented their final posters at the end of the semester in a symposium style, where they rotated between presenting to their peers and serving as an engaged audience. Co‐instructors graded this assignment in class using a rubric that assessed the scientific content, poster display, and delivery of the information (see S3).

## 3 METHODS FOR THE ASSESSMENT OF LEARNING GAINS

We evaluated our course using quantitative and qualitative methods. Quantitatively, students were asked to complete the classroom undergraduate research experience (CURE) surveys (Lopatto, 2008a), providing us with self‐reported course learning gains to quantify the course's effectiveness (Anaya, 1999).

All enrolled students anonymously and voluntarily completed the CURE survey at the beginning of the course (pre‐course) and another survey after the conclusion of the course (post‐course). This survey is a tool to evaluate traditional CURE courses and other science courses that incorporate research (Lopatto, 2008b). We used this survey because we had elements of the nature of science as well as communication skill sets incorporated into our course design (Lopatto, 2008b; see S1). The purpose of these surveys was to invite students to self‐report their learning gains and rate the elements of the course on a five‐point Likert scale (1 = smallest gain, 5 = largest gain). This course's CURE survey results were then compared with data from over nine thousand previous student responses to this same survey. Previously surveyed students came from various different ethnicities, attended public or private institutions across the country, and ranged from high school through graduate students. We also compared CURE responses with responses to the Survey of Undergraduate Research Experiences (SURE) responses in the cases where the same questions were provided (Lopatto, 2004). The students surveyed by SURE represented 41 universities and colleges, made up of equal amounts of males and females with eight different ethnicity groupings, and who ranged from first‐year to third‐year college students (SURE; Lopatto, 2004). The seven students in our CURE survey were all Caucasian, approximately equal in number of males and females, and were in either their second or third year at the University of Florida. Although the CURE survey rates learning gains in many areas, for the purpose of this paper we only present results pertinent to course goals and other non‐prioritized areas for comparison.

We planned a focus group interview to provide a forum for qualitative course evaluation. The anonymous focus group was moderated by an outside facilitator and included eight open‐ended questions written before the class began. The focus group discussion was then transcribed by a professional transcription service. The transcription was individually assigned open codes (Holstein & Gubrium, 2003) by two of the authors. After independently assigning open codes, the open codes were compared and consolidated. Upon consolidation, the open codes were placed into broader categories, which were used to help shape the discussion of this manuscript.

## 4 BENEFITS OF SCIENCE COMMUNICATION EDUCATION

### 4.1 Benefits to undergraduates

#### 4.1.1 Science communication skills

**Figure 1:**
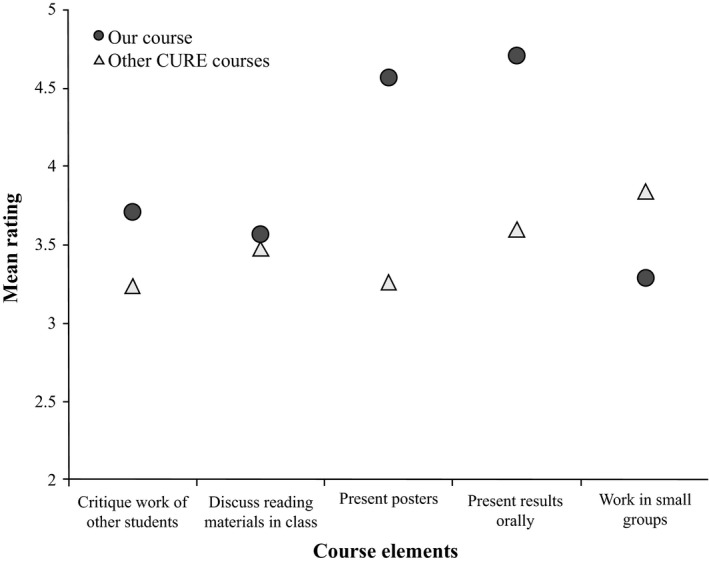
Self‐rated postcourse gains to *skills and abilities* reported by students. A rating of five is the highest gain and one is the lowest gain. The gray circles represent mean ratings of the students in the present study (our course, *n* = 7). For comparison, we show the overall mean gains reported by students (other CURE courses, *n* > 9,000) who took the CURE survey in 2014–2015, and who responded to the same CURE survey (white triangles)

**Figure 2:**
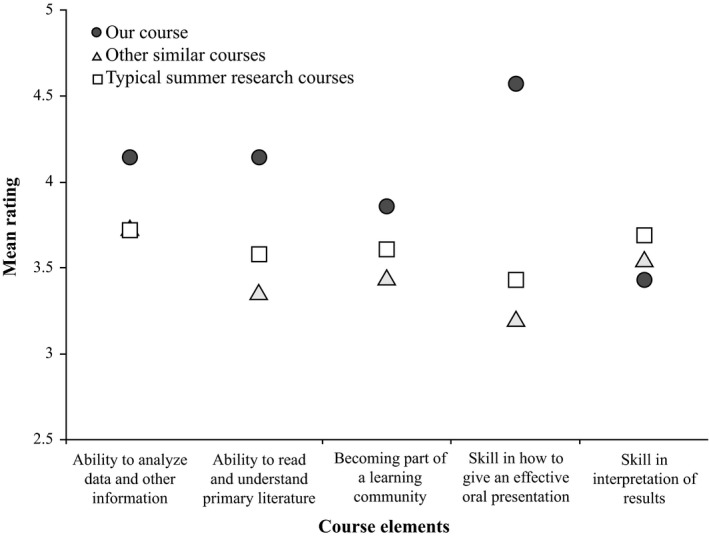
Self‐rated benefits to *development* reported by students after the course was completed. A rating of five is the highest gain and one is the lowest gain. The gray circles represent mean ratings of the students in the present study (our course, *n* = 7). For comparison, we show both the average learning gains reported by other students (similar courses, white triangles) enrolled in classroom research experience and by students who completed a summer laboratory research experience in 2014 and completed the Survey of Undergraduate Research Experiences (typical summer research course, white squares)

Students reported gains in two main categories: benefits to skills and abilities (Figure 1) and benefits to development as a scientist (Figure 2). Overall, our students reported high learning gains in communication as relevant to each category. The skills and abilities that were rated higher coincided with those required for science communication such as how to give an effective oral presentation (Figure 2). Additional non‐target areas, such as reading and understanding primary scientific literature, were also rated high (Figure 2).

#### 4.1.2 Confidence in delivering a science message

We found that teaching students science oral communication improved their confidence in speaking to various audiences. For example, one student said “personally, I've never presented someone else's research, let alone my own. So, even that little bit of experience was, I thought was, helpful… And I thought I developed my ability to present.” Another student remarked,

I think one of the best projects we've done in this course was the one‐minute summary of our research that had to be for a public audience. And I think that getting practice, […] presenting your research without all of the technical terms […] can go a long way toward science being better communicated toward the public.

Students received science communication knowledge and constructive feedback directly from senior researchers to help them make their science message stronger and more impactful. Students voiced that they recognized that complicated science messages can be ignored or even devalued by the public. We challenged students to bolster their critical thinking skills and build their confidence in oral communications through these assignments and class discussions (Figure 1). Our aim was to provide students with a foundation they can use to achieve excellence in science communication in the future. Ratings in some areas were not high relative to the national averages (Figures 1 and 2). These relatively low rankings were in areas that we did not prioritize in this iteration of the course; thus, they provide evidence that students were honestly reflecting on their learning gains specific to this experience.

### 4.2 Benefits to graduate student co‐instructors

Graduate students that were part of the teaching team and are authors on this paper benefitted from this experience. They gained higher‐level teaching and organizational skills that should raise their competitiveness for academic jobs (Cragg, Ramer, & Kramer, 2016; Walia & Sanders, 2016). For example, this course provided the opportunity to build a syllabus while thinking critically about class objectives, an experience that was new for most instructors. Graduate students also designed and implemented lessons that catered toward their class objectives. Additionally, graduate students improved their own communication skills through teaching others (for more information Cicirelli, 1972; Cortese, 2005). Strong professional relationships between graduate students and undergraduates were developed through this course that would not be possible in large lectures. Graduate students were also able to further their own research objectives by having undergraduates work on projects that were eventually published (e.g. Cirino & Miller, 2017; Emberts et al., 2016). Graduate students were able to assess the effectiveness of their teaching and mentorship through the use of the CURE survey and focus groups. The use of assessment tools to evaluate broader impacts is likely to become expected by more granting agencies in the future, and these students will be prepared. Importantly, the undergraduate students and the teaching team achieved another valuable end product from the class: research videos that were tailored to the public audience, helping with scientific outreach aims (see http://www.millerlab.net/).

## 5 CONCLUSION

The results from our interviews and surveys illustrate that we provided college students multiple opportunities to develop valuable and foundational skills in communication. Such experiences are often lacking in undergraduate research experiences (Coil et al., 2010; Mercer‐Mapstone & Kuchel, 2015). Students in our classroom were taught to recognize their audience, communicate science effectively to that audience, and evaluate the effectiveness of that message using multiple media. We were able to equip students with these skills that encouraged them to think more critically about their research and help them understand the importance of their work in a broader context while simultaneously supporting their growth as scientists in the classroom. Communication skills are among the top skills that employers are looking for in job candidates (Robles, 2012). The foundational skills provided through this course should benefit students in many career paths. Future studies should follow students as they move into their careers to assess benefits, if any, of this early training in science communication.

Our goal was to equip young undergraduate scientists with foundational science communication skills, while they simultaneously conducted novel research that met the research aims in the PI's laboratory. We achieved this goal with relatively little effort through an abbreviated summer semester. Our learning evaluations reveal that directing students to present their individual research projects in different ways to different audiences encouraged an early recognition of some of the challenges, and opportunities, that scientists face. Using an active learning classroom approach, we were able to meet national STEM goals (Handelsman & Brown, 2016) as well as our own established science communication goals. In addition, the course gave us an opportunity to interact in a new way with undergraduate researchers, providing a strong sense of unity and a team mentality.

## CONFLICT INTEREST

We have no competing interests.

## AUTHOR CONTRIBUTIONS

LAC, ZE, PNJ, PEA, and CWM all conceived, designed, and carried out the study. DL carried out the CURE survey analyses. ZE and PNJ carried out all focus group analyses. PEA generated figures from the CURE survey. LAC and CWM coordinated the study and drafted the manuscript. All authors gave final approval for publication.
