# Supplementary material for: Broadening the voice of science: Promoting scientific communication in the undergraduate classroom
Source: Ecol Evol. 2017 Oct 24;7(23):10124–30. doi: 10.1002/ece3.3501 (PMC5723632; doi:10.1002/ece3.3501)
Supplement: Supplementary file 1 [file ECE3-7-10124-s001.pdf]

The Nature of Science

ENY 4905 section 4087 Summer B 2015

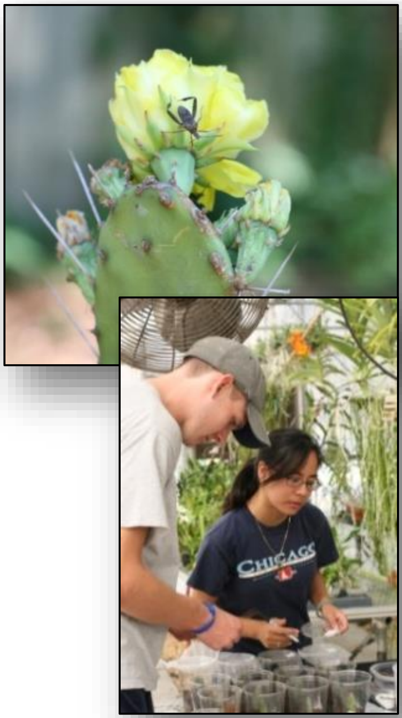

Location: Entomology and Nematology Department, Steinmetz Hall

Meeting time: Fridays 9:30 to 11am (Room 1027) and 11 – 12pm (see schedule for room number)

Instructors:

Dr. Christine Miller, Principle Investigator, cwmiller@ufl.edu
Pablo Allen, Ph.D. candidate, pabloallen@ufl.edu
Lauren Cirino, M.S. student, lacirino@ufl.edu
Zach Emberts, Ph.D. student, emberts@ufl.edu
Paul Joseph, M.S. student, pjoseph14@ufl.edu

Need help, have questions, or want some advice?

Pablo – posters and data
Paul – primary literature presentations, lab technique training
Zach or Lauren – Canvas, UF E-Learning, their facilitated topics
Dr. Miller – anything else

Course goals:

<u>Dedicated students will develop</u>

- An understanding of the scientific process through both classroom discussions and your laboratory research.
- An understanding of how living things interact with each other and their environment and demonstrate how this interaction drives biological change.
- Critical thinking skills to assess the relevance and importance of scientific findings.
- Recognition of the major challenges for conveying scientific findings to the general public
- Public presentation skills
- Science communication skills
- An understanding of ethical decision making in science
- Confidence in multiple lab techniques and basic knowledge in how to work with data

A major goal of this course is to visit, discuss, and analyze some of the fundamentals of the scientific pursuit and how science fits within our society and world. This course is designed for undergraduate students already involved in authentic research in the fields of ecology, evolution, or behavior. You can think about this course as a structured lab meeting with a large cohort of new researchers and ample access to more senior scientists.

Evaluation of learning/research accomplishment:

© UF UNIVERSITY of FLORIDA

Source of points	Points possible
“What is Science?” essay	20
Canvas weekly quizzes (4 quizzes, variable points)	30
Attendance and participation (5 weeks at 10pts/week)	50
Presentation of primary literature (including mandatory meeting with instructor prior to presentation)	50
Research Poster Presentation	100
Total	**250**

Grade and associated percent ranges %

**A**	**93-100**	**A-**	**90-92**	**B+**	**88-89**
**B**	**83-87**	**B-**	**80-82**	**C+**	**78-79**
**C**	**73-77**	**C-**	**70-72**	**D+**	**68-69**
**D**	**63-67**	**D-**	**60-62**	**E**	**<60**

Explanation of course activities and grading

- This is a “flipped classroom” course. We want to hear what you think! We expect you to invest sufficient time in preparing for class, then take the Canvas weekly quiz to test your learning. You will then come to class fully prepared to participate and learn from each other.
- You are expected to come to all classes, especially given the abbreviated schedule.
- You will be asked to explain to the other students what research you are involved with. This activity will encourage you to think about how your day-to-day activities link to the “big picture” of what your research is all about. Practice the art of science communication. Be compelling! Inspire awe!
- Students will work in pairs to present a paper from the scientific literature to the class.
- The research proposal presentation is an opportunity to practice presenting your research! Instructors will work with you to construct the poster. You are expected to also have a rehearsed presentation to go along with your dazzling poster. Think of your peers (other undergraduates) as your target audience.

Due dates are firm, unless you have a note from a physician or similar. If you know you have a conflict with something, inform us right away!

Brief schedule for topics and presentations. Please check Canvas for materials.

Date	Topic	Facilitator	Assignment or Quiz due on the Wednesday of that week?
**Week 1** July 3	No meeting (University holiday)		**yes (essay)**
**Week 2** July 10	What is science?	Dr. Miller	**yes**
	Lab techniques #1 (Room 3118)	Paul	
**Week 3** July 17	Evolutionary ecology and behavior	Lauren	**yes**
	Fun with data #1 (Room 1012)	Pablo	
**Week 4**	Scientific literacy and communication	Zach	**yes**
Monday, July 20	*Optional* lab techniques #2 in the Wayne lab, 417 Bartram, 11 to noon.	Paul	
July 24	Advanced Fun with data (Room 1012)	Pablo	
**Week 5** July 31	Science ethics	Lauren	**yes**
	Practice poster presentations for next week (Room 2216)	Pablo	
**Week 6** August 7	Student poster presentations	Paul	**no**

© UF UNIVERSITY of FLORIDA
