# Supplementary material for: Broadening the voice of science: Promoting scientific communication in the undergraduate classroom
Source: Ecol Evol. 2017 Oct 24;7(23):10124–30. doi: 10.1002/ece3.3501 (PMC5723632; doi:10.1002/ece3.3501)
Supplement: Supplementary file 2 [file ECE3-7-10124-s002.pdf]

Presentation Assessment

Student presentations from the primary scientific literature

You will receive up to 4 points for meeting with an instructor prior to presenting. To receive the 4 points, your presentation must be complete (addressing all points below), though some questions, issues, and concerns are fine.

Slides and content	Points Possible	Awarded
1) Title slide: presenter names, study title, author names, publication year, study journal, volume and page numbers.	2	
2) Introduction: presents the motivation for the study, the research question, and any hypotheses and predictions.	5	
3) Methods: clearly describes how, when, and where the study was conducted, introduces study system	5	
4) Results: summary of findings	5	
5) Discussion: present the author's interpretation of findings	5	

You will also be graded on your ability to give a presentation and communicate science *well*:

6) Clear evidence of being prepared and having rehearsed the talk. Speak clearly and confidently (watch um's, no gum!), and do not read the talk to the audience. Each student presents approximately an even portion of slides.	4	
7) Slides should be simple, clean, and easy to read *while we listen to you.* Listening to you is more important! Make text large and keep text to a minimum. No typos or grammatical errors. Avoid color combinations that make text hard to read (e.g, red on green).	4	
8) Use of appropriate photos, graphics, and/or diagrams. Please do not fill your presentation with unnecessary images, but please do use images when they will help clarify your story. Show a picture of the study organism. Do not overdo Powerpoint animations (spinning text, etc.)!	4	
9) Use of at least one figure from the manuscript to show results. You must orient the class to the figure and highlight any important trends.	4	
10) Presentation length: well-paced and does not run over time	4	
11) Question for the audience that is open-ended and likely to inspire critical thinking	4	

A few notes:

- Remember to set up the "big question" or "big problem" first. This is usually NOT specific to a single species, but instead describes a larger issue that the research in the paper addresses.
- **Minimize text on your slides**. Text and pictures are there to support what you are saying and to emphasize the major "take home" points, not to distract people from what you are saying.
- Practice, practice, practice. I generally practice a bit each day for 2 weeks before I give a talk at a conference. Giving a good talk is like acting. You may have a script, but you should sound natural and not like you are reading.
- Your question for the audience should be something non-technical, fun, or thought-provoking. Remember to get your audience thinking! The question, "so, what did you all think?" is not sufficient. If you are stumped, ask an instructor to help you out.
- The time limit for your presentation is 8 minutes with 2 minutes for questions. We will stop you from speaking at 8 minutes with a helpful signal at 7 minutes (so you know to finish up).
- The day of your presentation, please show up 10 minutes before class to load your presentation.

© UF UNIVERSITY of FLORIDA
