# Supplementary material for: Broadening the voice of science: Promoting scientific communication in the undergraduate classroom
Source: Ecol Evol. 2017 Oct 24;7(23):10124–30. doi: 10.1002/ece3.3501 (PMC5723632; doi:10.1002/ece3.3501)
Supplement: Supplementary file 3 [file ECE3-7-10124-s003.pdf]

Nature of Science
Summer 2015

Research Poster

**July 24th**	Deadline for the text and graphics for your posters. Upload to Canvas by 10pm as a Microsoft Word file. Any graphs and pictures should be embedded as enhanced metafiles, .tif, or .jpg. We will get back to you with comments as soon as possible. (10 points)
**July 31st**	Practice poster presentations in Room 2216 at 11am
**August 5th**	Complete poster materials due at 10pm as a pdf (25 points)
**August 7th**	Poster presentation to the class (65 points)

Students most commonly use Microsoft PowerPoint to design posters. Make sure you first set the page dimensions to the size of the poster you would like to print – the dimensions for this assignment should be up to, but not greater than, 36" x 36" square. Plenty of information is available on the internet regarding how to put together a compelling poster. Here are some of my tips:

1) A poster must strike a balance between providing technical information and sustaining interest. Here, your audience will be primarily undergraduates. So, make sure your poster is accessible. But, provide enough technical detail so that faculty and graduate students are convinced you have a great study idea.
2) Within a few seconds, the viewer should be able to identify the problem/question, what you are planning to do, your anticipated results, and why the research is important. Make it easy to grasp quickly.
3) Plan for short attention spans. For example, use headers that are more descriptive than just "methods", "results", and "discussion".
4) Too much text can be overwhelming. Preserve the meaning, but cut out details. One way to do this is to write out everything you want to convey, and then carefully reword sentences to be maximally informative while cutting out details.
5) Your poster is a guideline. An important part of the poster presentation will be what you have to say. Rehearse your presentation in advance. Practice on a roommate, friend, or family member.
6) Use space wisely. Remove empty space.
7) Look at other posters online and think about what makes some compelling and others boring. Here are some good sources: http://colinpurrington.com/tips/academic/posterdesign, http://www.flickr.com/groups/postersessions/

Components of your poster

Sections can have other names or even be lumped together. What is important is the content and order. The following issues need to be addressed:

- Introduction
    - Introduce the big question/problem and why it is important.
    - Explain how this study will fill the gap in knowledge
    - Introduce your study organism and explain why it is best for this question/problem

© UF | UNIVERSITY of FLORIDA

- Methods
  - Experimental design: must be well thought out so that it actually will address your study questions/problem
  - Provide enough detail on methods so that the viewer can see that your study is feasible
- Anticipated results
  - Graphs are required
  - Tables are optional. If used, they should be brief and clear
  - Any graphics that quickly convey take-home points are welcome
- Discussion/Summary/Conclusions
  - Briefly discuss the anticipated results
  - Remind everyone why the study is important
- Literature cited
  - Do not list these on the poster – provide them on paper to those who are interested (just a few copies will be fine)
  - At least 20 references. These should be the most salient, not just other similar studies
- Pictures
  - Provide a picture of your study species
  - You, at the proposed study site, or with the lab materials (you can get creative here)
  - Any other compelling photos

And, most important, have fun with this! Use it as an opportunity to think deeply about your research and improve your science communication skills.

<u>Other important information:</u>

- Please initially provide a one minute explanation of your research (like an elevator talk). Then, be prepared to either a) answer questions or b) provide an additional five minute presentation to explain the poster thoroughly.
- Don't just stop with a great poster – practice your presentation of the poster. Your target audience is undergraduates, so make sure it is clear, easy to understand, and free of jargon.

© UF UNIVERSITY of FLORIDA

Poster Presentation Evaluation Form

Based on the evaluation used for the Entomological Society of America
E=Excellent, **VG**= Very good, **G**=Good, **F**=Fair, **NI**=Needs improvement. Circle the appropriate score.

Presenter______________________________ Judge__________________________________

Scientific Content

	E	VG	G	F	NI
**Title clear and accurate**	5	4	3	2	1
**Research problem clearly stated**	5	4	3	2	1
**Context and importance of research demonstrated**	5	4	3	2	1
**Clear and concise materials and methods**	5	4	3	2	1
**Anticipated results clear and easily interpreted**	5	4	3	2	1
**Conclusions to point, corresponding to problem**	5	4	3	2	1
**Key terms defined and jargon avoided**	5	4	3	2	1
**Appropriate amount of details**	5	4	3	2	1
**Grammar, spelling, punctuation**	5	4	3	2	1
**Creativity in the scientific content**	5	4	3	2	1

Poster display

	E	VG	G	F	NI
**Overall appeal of display**	5	4	3	2	1
**Short text segments**	5	4	3	2	1
**Highlighting of major concepts**	5	4	3	2	1
**Logical order, minimum redundancy**	5	4	3	2	1
**Effective use of space**	5	4	3	2	1
**Legible with large fonts**	5	4	3	2	1
**Effective use of figures and/or tables, coordinated w/text**	5	4	3	2	1
**Useful and interesting photographs/illustrations**	5	4	3	2	1

Delivery

	E	VG	G	F	NI
**Short, to point presentation conveying "take home message"**	10	8	6	4	2
**Answers questions well; provides details as requested**	10	8	6	4	2
**Understandable to undergraduates; not confusing**	10	8	6	4	2

Required comments and advice for the presenter. What worked for you and what didn't? Was the proposed research interesting? Did he/she communicate it well? What advice do you have for this person for future presentations?

© UF UNIVERSITY of FLORIDA
